# Multisystem Inflammatory Syndrome in Children: Follow-Up of a Cohort from North India

**DOI:** 10.4269/ajtmh.21-0801

**Published:** 2022-02-16

**Authors:** Puspraj Awasthi, Vijay Kumar, Sanjeev Naganur, Karthi Nallasamy, Suresh Kumar Angurana, Arun Bansal, Rohit Kumar Manoj, Muralidharan Jayashree

**Affiliations:** ^1^ Division of Pediatric Critical Care, Department of Pediatrics, Advanced Pediatrics Centre, Chandigarh, India;; ^2^ Department of Cardiology, Postgraduate Institute of Medical Education and Research (PGIMER), Chandigarh, India

## Abstract

Multisystem inflammatory syndrome in children (MIC-S) is a hyperinflammatory manifestation of severe acute respiratory syndrome coronavirus-2 (SARS-CoV-2) infection. Information on the long-term outcome of MIS-C is limited. This study was conducted to describe the long-term outcome of MIS-C from a tertiary care center in North India. Children admitted with MIS-C from September 2020 to January 2021 were followed up after discharge until June 2021. The details during the acute phase (clinical features, investigations, treatment, and outcome) and follow-up (symptoms, echocardiographic findings, ongoing treatment, and outcome) were collected retrospectively. During the acute phase, 40 children presented at median (interquartile range [IQR]) age of 7 (5–10) years with fever, mucocutaneous, gastrointestinal, and respiratory symptoms. The majority (66.7%) of the children had positive SARS-CoV-2 serology and elevated inflammatory markers (C-reactive protein, procalcitonin, ferritin, D-dimer, and fibrinogen), lymphopenia, and thrombocytopenia. Eighty percent had shock, 72.5% had myocardial dysfunction (left ventricular ejection fraction <55%), and 22.5% had coronary artery dilatation or aneurysm. Treatment included pediatric intensive care unit admission (85%), intravenous immunoglobulin (100%), steroids (85%), aspirin (80%), vasoactive drugs (72.5%), and invasive mechanical ventilation (22.5%). Two (5%) children died because of refractory shock. Thirty-four children were followed up with until a median (IQR) of 5 (3–6) months. During the follow-up, a majority were asymptomatic, myocardial function returned to normal in all, and only one had coronary artery aneurysm. Prednisolone and aspirin were given for a median (IQR) of 3 (2–4) weeks and 4 (4–6) weeks after discharge, respectively. There was one readmission and no death during the follow-up. To conclude, the long-term outcome of MIS-C is generally favorable with resolution of cardiovascular manifestations (myocardial dysfunction and coronary artery changes) in the majority of children during follow-up.

## INTRODUCTION

Multisystem inflammatory syndrome in children (MIS-C) is an uncommon postinfectious hyperinflammatory manifestation of severe acute respiratory syndrome coronavirus-2 (SARS-CoV-2) infection in children. ^1^^,^ ^2^ It is characterized by fever, mucocutaneous features (conjunctivitis, strawberry tongue, cervical lymphadenopathy, rash, and peripheral limb edema), gastrointestinal (GI) involvement (pain abdomen, vomiting, and diarrhea), cardiovascular manifestations (myocardial dysfunction, coronary artery changes, and shock), raised inflammatory markers (C-reactive protein [CRP], erythrocyte sedimentation rate [ESR], procalcitonin, ferritin, interleukin-6 [IL-6]), and evidence of an epidemiological link to SARS-CoV-2 infection in recent past (positive SARS-CoV-2 reverse transcriptase polymerase chain reaction [RT-PCR] or serology, contact with a case of SARS-CoV-2 infection, or symptoms suggestive of SARS-CoV-2 infection in the last 2–6 weeks). ^1^^,^ ^3^^–^ ^5^

A significant proportion of children with MIS-C have cardiovascular manifestations in the form of myocardial dysfunction, shock, coronary artery dilatation and aneurysms, pericardial effusion, and rhythm disturbances. ^3^^–^ ^7^ With supportive treatment and immunomodulation (intravenous immunoglobulin [IVIG] and steroids), the short-term outcome is good and mortality is 0–2%. ^3^^–^ ^8^ Long-term treatment with glucocorticoids, aspirin, or anticoagulants in MIS-C is based on experience with other inflammatory conditions like Kawasaki disease (KD) and the exact duration of treatment is not known. The suggested duration for immunomodulation is 2–3 weeks or even longer with severe disease. ^9^ Whether the myocardial dysfunction and coronary artery changes are transient or have long-term impact on the health of these children is yet to be elucidated. Information on the long-term outcome of MIS-C is limited to a few studies from developed countries. ^10^^–^ ^16^ Therefore, we planned this follow-up study to describe the clinical features, echocardiographic findings, and long-term outcome of children with MIS-C from a tertiary care center in North India.

## METHODOLOGY

This retrospective study uses the follow-up data of a cohort of children < 12 years admitted with the diagnosis of MIS-C between September 2020 and January 2021 to a tertiary care hospital in North India. ^4^ These children were followed up with until June 2021 and the follow-up data were collected. The study protocol was approved by the Institute Ethics Committee and the final manuscript was approved by the Departmental Review Board.

The details of methodology for data collection and management during the acute phase is described elsewhere. ^4^ Briefly, demographic and clinical details; laboratory investigations including inflammatory markers; cardiovascular involvement (myocardial dysfunction, coronary artery dilatation or aneurysm, and shock); details of treatment (pediatric intensive care unit [PICU] admission, invasive mechanical ventilation, vasoactive drugs, IVIG, steroids, and immunomodulators); and short-term outcome (mortality and length of hospital stay) were gathered from the case files. The diagnosis of MIS-C was established on the basis of case definition put forth by the WHO (https://www.who.int/news-room/commentaries/detail/multisystem-inflammatory-syndrome-in-children-and-adolescents-with-covid-19). The diagnosis of current or recent SARS-CoV-2 infection was confirmed by nasopharyngeal swab SARS-CoV-2 RT-PCR, or serology, or contact with a case with SARS-CoV-2 infection in recent past (2–6 weeks prior). The management protocol we followed is published elsewhere ^17^ and was based on the recent guidelines for managing MIS-C. ^9^^,^ ^18^^,^ ^19^ Along with supportive and intensive care, mild cases were treated with IVIG (2 gm/kg over 12–24 hours, maximum dose 100 gm) alone or in combination with low-dose steroids (methylprednisolone, 1–2 mg/kg/day, max 60 mg/day). Cases with moderate to severe disease were treated with IVIG and high-dose methylprednisolone (10–30 mg/kg/day, maximum dose 1,000 mg/day, for 1–3 days) followed by low-dose steroids (methylprednisolone or prednisolone, 1–2 mg/kg/day, maximum dose 60 mg/day, continued and tapered slowly over 2–3 weeks or even longer for severe disease). Low-dose aspirin (3–5 mg/kg/day, maximum dose 81 mg, once a day) was used in all cases with MIS-C (unless contraindicated by the presence of active bleeding, significant risk of bleeding, and/or a platelet count ≤ 80,000/µL) and continued for 4 to 6 weeks. Discharge criteria included clinically stable child with no fever, decreasing inflammatory markers, and stable cardiac function without vasoactive drugs for more than 48 hours. ^17^

In this study, the children with MIS-C were followed up with at 1–2 weeks and 4–6 weeks after discharge by the treating team and pediatric cardiologist. At each follow-up visit, a detailed clinical evaluation and echocardiography were performed. The ongoing treatment in form of glucocorticoids, aspirin, or any other treatment and its duration was noted. Later follow-ups (every 1–2 months) were done either in the physical outpatient department (OPD) or via telephone until June 2021. In addition, telephone consultations were held with those who did not come for an in-person follow-up visit. All children with abnormal echocardiography or electrocardiography (ECG) during the acute phase were followed up with in person in the OPD. Myocardial dysfunction was defined as left ventricular ejection fraction (LVEF) < 55%. Coronary artery dilatation or aneurysm was defined as coronary artery diameter ≥ 2 z score and ≥ 2.5 z score, respectively, and giant aneurysm as ≥ 10 z score or ≥8 mm. The data were collected from the follow-up records on a predesigned study proforma and included age, sex, duration of follow-up, any clinical symptoms, ECG findings, and echocardiographic findings (LVEF and coronary artery diameter). The ongoing treatment in the form of steroids, aspirin, anticoagulation, and its duration were recorded.

### Statistical analysis.

Appropriate data entry and statistical analysis were performed in Microsoft Excel 2010 (Microsoft, Redmond, WA) and SPSS software version 20 (SPSS, Inc, Chicago, IL). Descriptive statistics (median, interquartile range [IQR], number, and percentages) were used for describing various follow-up variables.

## RESULTS

### Acute phase.

The details of clinical features, laboratory investigations, treatment, and outcome of 40 children with MIS-C are reported elsewhere. ^4^ Briefly, the most common clinical features were fever and mucocutaneous, GI, and respiratory symptoms. The evidence of current or recent SARS-CoV-2 infection was noted in the form of positive SARS-CoV-2 serology (66.7%), positive SARS-CoV-2 RT-PCR (10%), and history of exposure to a SARS-CoV-2 case (12.5%) (Table 1). The most common laboratory abnormalities noted were elevated N-terminal-pro B-type natriuretic peptide, CRP, D-dimer, ferritin, fibrinogen, and procalcitonin; and lymphopenia and thrombocytopenia. ^4^ Common echocardiographic findings were LVEF < 55% (72.5%) and coronary artery dilatation or aneurysm (22.5%). Treatment included PICU admission (85%), invasive mechanical ventilation (22.5%), vasoactive drugs (72.5%), IVIG (100%), steroids (85%), and aspirin (80%). The mortality was 5% (*N* = 2), and both of these children died within 24 hours of admission because of refractory shock. ^4^ During the acute phase, four (10%) cases had hemorrhagic nonpurulent conjunctivitis, ^20^ two (5%) had digital gangrene, ^21^ one (2.5%) had transient arrhythmias (junctional rhythm) during acute illness that recovered spontaneously, and one (2.5%) had neuromuscular weakness attributed to critical illness polyneuropathy.

**Table 1:** Clinicolaboratory profile, treatment, and outcome of children with MIS-C during acute phase (*N* = 40)

Characteristics	Total cases, *N* = 40
Male, *n* (%)	26 (65)
Age (years), median (IQR)	7 (5–10)
Clinical features	
Fever, *n* (%)	39 (97.5)
Mucocutaneous features, *n* (%)	32 (80)
Abdominal symptoms, *n* (%)	29 (72.5)
Respiratory symptoms, *n* (%)	20 (50)
Hemorrhagic nonpurulent conjunctivitis, *n* (%)	4 (10)
Digital gangrene, *n* (%)	2 (5)
Duration of illness (days), median (IQR)	6 (5–7)
Laboratory investigations	
Lymphopenia, *n* (%)	26 (65)
Thrombocytopenia, *n* (%)	20 (50)
Elevated CRP, *n* (%)	38 (95)
Elevated procalcitonin, *n* (%)	34 (80)
Elevated ferritin, *n* (%)	36 (90)
Elevated D-dimer, *n* (%)	37 (92.5)
Elevated fibrinogen, *n* (%)	35 (87.5)
Elevated NT-proBNP, *n* (%)	40 (100)
Confirmation of exposure	
Positive SARS-CoV-2 serology, *n* (%)	20/30 (66.7)
Positive SARS-CoV-2 RT-PCR, *n* (%)	4 (10)
Contact with positive case of SARS-CoV-2, *n* (%)	5 (12.5)
Echocardiography performed	40 (100)
LVEF at admission, median (IQR)	45 (36–50)
Myocardial dysfunction (LVEF < 55%), *n* (%)	29 (72.5)
Coronary artery dilatation or aneurysm, *n* (%)	9 (22.5)
Treatment and outcome	
Admitted to PICU, *n *(%)	34 (85)
Invasive ventilation, *n* (%)	9 (22.5)
Shock, *n* (%)	32 (80)
Vasoactive drugs, *n* (%)	29 (72.5)
IVIG, *n* (%)	40 (100)
Steroids, *n* (%)	34 (85)
Aspirin, *n* (%)	32 (80)
Mortality, n (%)	2 (5)

CRP = C-reactive protein; IQR = interquartile range; IVIG = intravenous immunoglobulin; MIS-C = multisystem inflammatory syndrome in children; NT-proBNP = N-terminal-pro B-type natriuretic peptide; PICU = pediatric intensive care unit; RT-PCR = reverse transcriptase polymerase chain reaction; SARS-CoV-2 = severe acute respiratory syndrome coronavirus-2.

### Follow-up.

**Figure 1.**
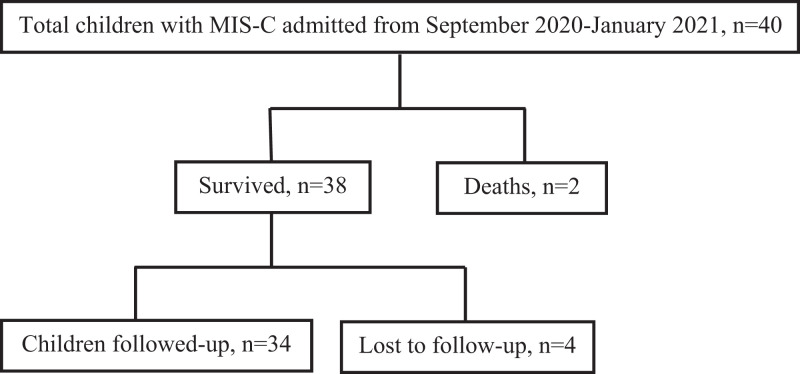
Study flow diagram.

Two children died during the acute phase, and four did not come for follow-up. The remaining 34 children were followed up with for a median (IQR) duration of 5 (3–6) months after discharge (Figure 1). The median (IQR) age at the last follow-up was 7.4 (5.3–10) years. Children who did not come for follow-up (*N* = 4) were asymptomatic as per their telephone consultations.

During follow-up, four (11.8%) children reported one or more symptoms (palpitations, neuromuscular weakness, nonspecified febrile illness, and rapid breathing and wheezing in one each) (Table 1). The child with palpitations was admitted for 48 hours, and her ECG showed sinus tachycardia with ST segment elevation in the left-side chest leads, but this resolved on its own within 24 hours, and the echocardiography at that time was normal. One child developed neuromuscular weakness during the acute phase that persisted during the follow-up. During the acute phase, he had shock and myocardial dysfunction and required prolonged invasive mechanical ventilation and vasoactive drugs. He also received high-dose steroids for 3 days. The PICU course was prolonged. A nerve conduction study showed axonal neuropathy. Neuromuscular weakness was attributed to the critical illness polyneuropathy. The clinical course improvement during the subsequent follow-up with physiotherapy and rehabilitation. Four (10%) children had hemorrhagic nonpurulent conjunctivitis during the acute phase that resolved within 5–21 days. The clinical pictures are reported elsewhere. ^20^ Two children developed peripheral digital gangrene bilaterally on their feet during the acute phase (day 3 and day 7 of hospitalization). Both children had positive SARS-CoV-2 serology, negative RT-PCR, and shock requiring vasoactive drugs. Only one had myocardial dysfunction. Both children received low-molecular-weight heparin (1 mg/kg, 12-hourly, subcutaneously) in addition to IVIG, glucocorticoids, and aspirin. In one child, digital gangrene resolved completely within 2–3 weeks, and the other developed autoamputation of a few toes in the subsequent 4–6 weeks. Both children were followed up with until 3 months after discharge. The clinical pictures are reported elsewhere. ^21^ All children received oral prednisolone and aspirin for a median duration of 3 (2–4) weeks and 4 (4–6) weeks, respectively (Table 2). There were no side effects related to prednisolone or aspirin.

**Table 2:** Profile of children with MIS-C during follow-up (*N* = 34)

Characteristics	Follow-up, *N* = 34
Age in years, median (IQR)	7.4 (5.3–10)
Males, *n* (%)	22 (64.7)
Duration of follow-up in months, median (IQR)	5 (3–6)
Signs and symptoms, *n* (%)	4 (11.8) Palpitation in 1 Neuromuscular weakness in 1 Nonspecified febrile illness in 1 Rapid breathing and wheezing in 1
Resolution of hemorrhagic nonpurulent conjunctivitis	Resolved in all by 5–21 days
Resolution of digital gangrene	Resolved in 1 Digital autoamputation in 1
Abnormal ECG, *n* (%)	1 (2.9)
Echocardiography done, *n* (%)	34 (100%)
Ejection fraction, median (IQR)	60 (55–65)
Myocardial dysfunction, *n* (%)	2 (5.9) in first follow-up of 2 weeks 0 in subsequent follow-ups
Coronary artery dilation or aneurysm, *n* (%)	1 (2.9)
Duration of Prednisolone in weeks, median (IQR)	3 (2–4)
Duration of Aspirin in weeks, median (IQR)	4 (4–6)
Readmission, *n* (%)	1 (2.9)
Mortality, *n* (%)	0

ECG = electrocardiography; IQR = interquartile range; MIS-C = multisystem inflammatory syndrome in children.

During follow-up, echocardiography showed mild left ventricular hypokinesia in 2 (5.9%) children on the first follow-up visit at 2 weeks, but this normalized at the subsequent follow-up visit at 4 weeks. Left ventricular ejection fraction was 60% (55–65%) during the last follow-up of 5 (3–6) months after discharge. Coronary artery dilatation or aneurysm was noted in 22.5% (*N* = 9) during the acute phase. During the follow-up, only one child had coronary artery dilatation, which persisted until 8 months after discharge with a right coronary artery diameter of 4 mm (+3.3 z score). This child was continued on aspirin. There was one readmission after discharge (described earlier) and no mortality during follow-up (Table 2).

## DISCUSSION

The short-term outcome of MIS-C is excellent with supportive care and immunomodulation with low mortality (< 2%). ^3^^–^ ^6^ However, information on the medium- and long-term outcomes of MIS-C is limited, especially with regard to cardiac outcomes (myocardial dysfunction and coronary artery changes). Treatment of the acute phase and long-term management of MIS-C are based on experience with other inflammatory conditions like KD. The most commonly used immunomodulatory strategies are IVIG and steroids. The exact duration of long-term treatment is not known. However, immunomodulation has been suggested for 2–3 weeks or even longer for severe disease. ^9^^,^ ^17^ The mortality of 5% (*N* = 2) in this index study is higher than that reported from developed countries. ^3^^–^ ^8^ Both cases died because of refractory shock within 24 hours of admission. This highlights the importance of early identification, resuscitation (with fluids and vasoactive drugs), and targeted immunomodulation upon first contact with the healthcare facility. ^4^

The majority of studies reporting long-term follow-up of children with MIS-C are from developed countries, ^10^^–^ ^16^ and there is lack of such data from the Indian subcontinent. In this retrospective study from North India, we described the follow-up of 34 children with MIS-C who were admitted during the first wave of SARS-CoV-2 infection and demonstrated that majority of the children were asymptomatic and myocardial dysfunction and coronary artery changes resolved in majority during follow-up. Similarly, Penner et al., ^10^ in a retrospective study, described follow-ups with 46 children (< 18 years) with MIS-C up to 6 months after discharge and noted that systemic inflammation resolved in all except one, 90% had positive SARS-CoV-2 IgG antibodies, 87% were free of GI symptoms, 96% had normal echocardiography, and only two had persistent coronary artery dilatation. Feldstein et al. ^12^ reported a cohort of 539 children with MIS-C and demonstrated that during the acute phase, left ventricular dysfunction and coronary artery aneurysm were noted in 34% (172/503) and 13% (57/424) cases, respectively, which normalized in 99% and 100% of the children within 90 days, respectively. These findings were more or less similar to the index study. Farooqi et al. ^16^ reported 45 children (< 21 years) with MIS-C from New York who were followed up with for 5.8 (1.3–6.7) months. During the acute phase, a majority had significant inflammation, generalized lymphopenia, thrombocytopenia, moderate to severe echocardiographic abnormalities (44%), and coronary artery dilatation (9%). By 1–4 weeks, inflammatory markers normalized, only 18% had mild echocardiographic findings, and all had normal coronary arteries. By 4 to 9 months, only one had mild myocardial dysfunction. During the acute phase of MIS-C, left ventricular dysfunction (35–70%) and coronary artery dilatation or aneurysm (9–16%) are common, ^3^^,^ ^4^^,^ ^10^^–^ ^16^ which has raised concern over the long-term outcome of MIS-C. However, results of available follow-up studies demonstrated that despite significant cardiovascular involvement (myocardial dysfunction, shock, and coronary artery changes) during the acute phase, left ventricular dysfunction and coronary artery dilatation or aneurysm resolved rapidly in the majority of the children, and there was no mortality. ^10^^–^ ^16^ This suggest that myocardial involvement during the acute phase is possibly because of stunning or edema of the myocardium followed by rapid restoration of the myocardial function rather than inflammatory myocardial damage. This is in contrast to progressive endovascular changes and inflammatory damage seen in other inflammatory conditions such as KD. ^22^^–^ ^24^

The strengths of this study include the long duration of follow-up. The detailed echocardiography was done by a pediatric cardiologist during follow-up to look for any residual myocardial dysfunction and coronary artery changes. Limitations of this study include that it was a single-center study with a relatively small sample size. The retrospective nature of study limited the uniformity of the follow-up data. A possibility of referral bias cannot be ruled out, as children with greater severity of illness being referred to us and majority (85%) of children in index study required admission to PICU. Adolescents with MIS-C were not included, as our center only admits children < 12 years of age. An assessment of the physical, mental, emotional, social, and psychosocial status by using objective scales was not performed. The inflammatory markers and SARS-CoV-2 serology were not measured during the follow-up.

MIS-C is a rare complication of SARS-CoV-2 infection with significant inflammation and cardiovascular dysfunction during its acute phase. Limited data showed that myocardial dysfunction and coronary artery changes resolved rapidly with the suggested treatment. Long-term prospective studies involving multiple centers are needed to validate the findings of this index study and to describe the long-term outcome and natural history of MIS-C, especially cardiovascular outcomes.

## CONCLUSION

Although MIS-C is characterized by multisystemic severe disease during its acute phase, the long-term outcome is generally favorable. The cardiovascular manifestations (myocardial dysfunction and coronary artery changes) resolved in majority of the children during follow-up. The outcomes reported in this study will guide healthcare providers about the natural history of MIS-C.
